# Review of Solar, Thermal, and Electromagnetic Energy Harvesting for Satellites

**DOI:** 10.3390/s26134254

**Published:** 2026-07-04

**Authors:** Yurui Lu, Rongke Gao, Xiaozhe Chen, Lu Wang

**Affiliations:** 1 College of Control Science and Engineering, China University of Petroleum (East China), Qingdao 266580, China; 2305020121@s.upc.edu.cn (Y.L.); chenxiaozhe@upc.edu.cn (X.C.); 2 School of Instrument Science and Technology, Xi’an Jiaotong University, Xi’an 710049, China

**Keywords:** energy harvesting, satellite, photovoltaic, radio frequency, thermoelectric

## Abstract

With the rapid development of commercial aerospace, emerging applications such as satellite constellations, space-based communications, and orbital computing platforms have significantly increased the demand for efficient and reliable spacecraft power systems. Abundant exploitable energy exists in the space environment, including Air Mass Zero (AM0) solar radiation, spacecraft surface temperature gradients, ambient electromagnetic radiation, and radioisotope thermal energy, making multi-source energy harvesting a promising approach for improving satellite energy autonomy and system redundancy. This paper reviews the following four key space energy harvesting technologies: photovoltaic power generation, radio frequency (RF) energy harvesting, thermoelectric energy harvesting, and radioisotope thermoelectric generators (RTGs). The impacts of harsh space environmental factors on device performance and reliability are analyzed, and the applicability of different technologies in low Earth orbit (LEO), geostationary orbit (GEO), and deep-space missions is discussed. Furthermore, a multi-source self-powered satellite energy architecture integrating energy harvesting, energy storage, and power management is proposed. Finally, the major challenges and future development trends of satellite energy harvesting systems are summarized.

## 1. Introduction

In recent global commercial aerospace development, new application scenarios such as low-orbit constellation deployment, satellite communication foundation, and space-based computing nodes have emerged. As the scale of constellations continues to expand, the energy demand of satellites grows accordingly [1,2]. Many new energy and aerospace enterprises are dedicated to researching and developing space-efficient photovoltaic components, radiation-resistant materials, and lightweight energy subsystems. Space photovoltaic and orbital energy systems are key parts of future space infrastructure. When it comes to high-frequency commercial aerospace launches and large-scale network deployment, the reliability, redundancy, and long-term stable power supply capacity of the energy system are the key to mission success. Although traditional spacecraft power systems based on photovoltaic power generation and rechargeable batteries are still mainstream, in order to cope with the complex and ever-changing conditions in the universe, such as spacecraft operating in environments without sunlight or the failure of a single power-generation method, exploring multi-energy coordination and intelligent control mechanisms at the system level is of great significance for engineering.

The stable operation of spacecraft takes the energy system as the core infrastructure, which greatly prolongs the operating life. “Dongfanghong I” uses a disposable chemical battery and only works for 28 days. Voyager 1 has been in operation for 48 years, relying on a radioisotope thermoelectric generator (RTG) [3]. The current satellite power system combines photovoltaic arrays with rechargeable batteries. Although this technology is mature and reliable, it has limitations in complex orbits and high-power missions. Eclipses cause intermittent power supply, high-energy particle radiation leads to performance degradation, micro-satellite platforms have mass and volume limitations, and there is a continuous increase in power requirements for communication payloads and control systems.

Deep space exploration, distributed satellite networks and long-term unmanned missions are continuously advancing. A single photovoltaic power supply mode is difficult to meet the requirements of high reliability and redundancy. There are various potential energy sources in the space environment itself.

Space photovoltaic systems typically provide power ranging from several watts for CubeSats to over 100 kW for large space platforms. Solar PV power generation relies on the photoelectric effect and the built-in field in the p-n junction for carrier separation. When the photon energy exceeds the semiconductor bandgap, electron-hole pairs are generated and separated by the built-in electric field, producing electrical current. Multi-junction cells employ bandgap engineering for spectral-selective absorption, improving quantum efficiency and reducing thermal losses, making them a key technology for high-efficiency space power systems.

Radio frequency energy harvesting relies on the coupling between electromagnetic waves and conductors as well as rectification technology. It has low-power output, typically ranging from microwatts to milliwatts, which is several orders of magnitude lower than that of space photovoltaic systems and insufficient to support primary spacecraft loads; therefore, it is mainly used as an auxiliary power source for low-power circuits, sensors, and micro-/nanosatellites. Alternating current is induced in the antenna and converted into direct current through nonlinear rectifiers such as Schottky diodes or two-dimensional material rectifiers. The key is to improve the rectification efficiency and impedance matching at low-power density. The design of wideband antennas and low-threshold rectification structures remains a major research focus.

Thermoelectric generators use the Seebeck effect, and the output power ranges from milliwatts to kilowatts. Radioisotope thermoelectric generators (RTGs) are suitable for deep space probes, and they have the characteristics of reliability and high output. The temperature difference across the thermoelectric material causes carrier diffusion, generating a potential difference, and directly converting thermal energy into electrical energy. The efficiency depends on the ZT value of the material (ZT = *S*^2^*σT*/*κ*), where *σ* is the electrical conductivity, S is the Seebeck coefficient, and κ is the thermal conductivity. Methods to improve the performance of space thermoelectric devices include increasing phonon scattering and band structure engineering, reducing lattice thermal conductivity and improving the power factor.

Existing review articles mainly focus on individual technologies. For example, research advances in thin-film solar cells [4,5,6], perovskite solar cells [7] and its application in space [8]. However, comparative evaluations of multiple space energy harvesting technologies under different orbital environments and mission requirements remain limited. To address this gap, this review provides a unified discussion of photovoltaic, RF, thermoelectric, and radioisotope-based energy systems, emphasizing their environmental adaptability, technological maturity, and applicability to LEO, GEO, and deep-space missions.

This review summarizes recent advances in satellite energy harvesting technologies based on a comprehensive survey of the literature published between 2015 and 2026. Relevant publications were collected from major scientific databases and repositories, including Web of Science, IEEE Xplore, ACS Publications, Nature Portfolio, ScienceDirect, MDPI, NASA Technical Reports Server, Scopus, and SpringerLink. The selected literature covers photovoltaic, RF energy harvesting, thermoelectric, and radioisotope power technologies, as well as their performance under the space environment. The collected studies were analyzed and compared to identify current development trends, technical challenges, and future research opportunities for satellite power systems.

**Figure 1:**
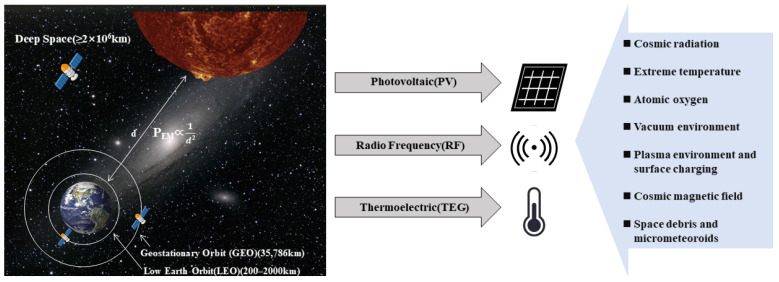
Schematic diagram of satellite energy harvesting.

Figure 1 shows the idea of this article. Three spacecraft with different orbits generate electricity through different methods. The solar irradiance available to photovoltaic systems decreases approximately according to the inverse-square law with increasing distance from the Sun. The space environment on the right represents the impact on the satellite’s self-powered system.

## 2. Energy Harvesting Methods

The energy in outer space includes that from the sun and the energy recovered by the spacecraft itself. This review focuses on four types of energy harvesting, namely solar photovoltaic power generation, electromagnetic RF power generation, thermoelectric power generation, and radioisotope thermoelectric generators.

### 2.1. Solar Photovoltaic Power Generation

Satellite solar photovoltaic power generation belongs to a common and mainstream situation. When people mention satellites, they will associate with their solar panels. Next, the photovoltaic technology on satellites will be elaborated in detail.

#### 2.1.1. Silicon Solar Cells

Space silicon solar cells are primarily based on BSF (back surface field) crystalline silicon technology. Owing to their mature manufacturing process, low cost, and proven reliability, they were widely employed in early spacecraft. However, BSF technology can no longer meet today’s needs. With continuous technological changes and developments, PERC (Passivated Emitter and Rear Cell), HJT (Heterojunction with Intrinsic Thin-layer), IBC (Interdigitated Back Contact), and TOPCon (Tunnel Oxide Passivated Contact) have become the mainstream silicon-based solar cell technologies. Most of these technologies are still in the laboratory research stage (TRL 3). This paper reviews these promising advanced photovoltaic technologies, hoping that they can contribute to future satellite photovoltaic power generation.

**Figure 2:**
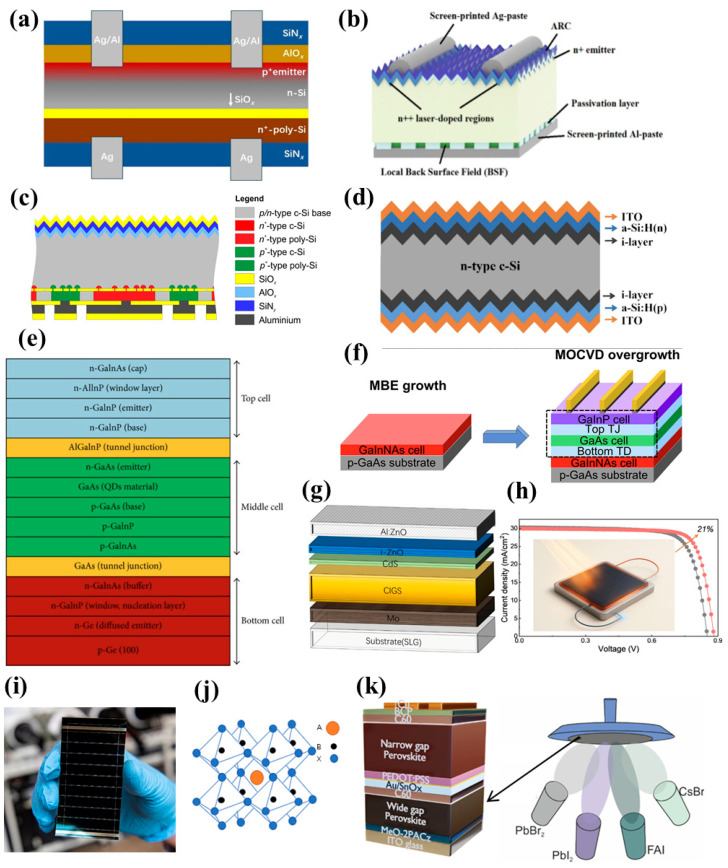
Composition of solar cells with different materials. (**a**) TOPCon silicon solar cells [56]. (**b**) PERC silicon solar cells [57]. (**c**) POLO-IBC silicon solar cells [15]. (**d**) HJT silicon-based solar cells [10]. (**e**) The structure of III/-V-based solar cells [58]. (**f**) GaInP/GaAs/GaInNAs solar cells [23]. (**g**) CIGS solar cells [59]. (**h**) CdSeTe thin-film solar cells [60]. (**i**) Appearance of perovskite solar cells (photographed by Dennis Schroeder/National Renewable Energy Laboratory). (**j**) Schematic diagram of perovskite crystal cell. (**k**) Structure of perovskite solar cells [61].

The silicon heterojunction (SHJ) structure is formed by depositing intrinsic amorphous silicon (i—a—Si:H) and p-type/n-type amorphous silicon (p/n—a—Si:H) on an n-type silicon wafer. It has a symmetric heterojunction structure with high efficiency, low degradation, and high double-sided power generation efficiency. In addition, the solar cells using the HJT technology also have excellent radiation tolerance. Several space agencies have completed the calibration of space irradiation, and small satellites are undergoing in-orbit verification. This is a promising solution for future low-cost photovoltaic cells. However, the cost of HJT technology is still relatively high. Yu et al. [9] used undoped SnO_2_ as a transparent electrode to prepare SHJ solar cells, and the conversion efficiency reached 25.2%. Traditional TCO contains rare elements such as indium (In) and also needs to be doped. In this study, a dense and ultra-thin SnO_2_ layer is prepared by ALD. This layer has good conductivity and high light transmittance and enhances the collection of carriers by band alignment. This work shows that materials containing earth elements can completely replace expensive ITO, opening up a new way to reduce the cost of high-efficiency silicon-based solar cells. Razzaq et al. [10] studied the ultraviolet stability of perovskite/heterojunction (com HJT) solar cells/modules. The amorphous silicon passivation layer in HJT cells is sensitive to ultraviolet, long-term exposure or lack of protection. The authors compared different encapsulation materials and ultraviolet coatings and analyzed the changes in chemical bonds in the microstructure of the amorphous silicon layer under ultraviolet irradiation. The study proposed a strategy for improving integrated encapsulation, blocking the damage of high-energy photons to the passivation interface, and improving the long-term reliability of HJT modules outdoors. Figure 2c presents the structure of a silicon HJT solar cell.

PERC technology is to add an Al_2_O_3_ passivation layer on the back of the Al–BSF solar cells, form local contacts through laser ablation, and use the high negative charge density of Al_2_O_3_ to passivate the back surface field and reduce recombination losses. Its radiation resistance is weak, and its application in space needs to be verified. Allen et al. [11] carried out research on “passivated contact” in silicon solar cells. The results show that the traditional PERC solar cells are close to the theoretical limit, and more efficient improvement depends on minimizing carrier recombination at metal contacts. The author analyzes the carrier-selective contact structures such as passivated emitter and rear contact (TOPCon), explains the physical mechanisms by which they achieve high open-circuit voltage and low contact resistance. Additionally, they conclude that passivated contacts are the key to silicon photovoltaic technology having an efficiency exceeding 26%. Figure 2b presents the structure of a silicon PERC solar cell.

N-type TOPCon replaces the rear dielectric passivation and local Al contact of PERC with an ultra-thin tunneling oxide (SiO_2_, 1–2 nm) and doped polysilicon stack, where SiO_2_ provides surface passivation and enables carrier tunneling, and the polysilicon layer acts as a conductive and passivating contact, forming a full-area “passivation-contact” structure without laser ablation. Recent studies have revealed the atomic-scale mechanism responsible for the high efficiency of TOPCon solar cells. By directly observing the tunnel oxide interface using aberration-corrected transmission electron microscopy, researchers identified passivating pinholes that simultaneously enable carrier tunneling and suppress interface recombination. Based on this optimized passivation-contact architecture, industrial-scale TOPCon solar cells achieved a certified conversion efficiency of 25.4% with an open-circuit voltage of 738.7 mV, demonstrating the significant potential of TOPCon technology for next-generation high-efficiency silicon photovoltaics. Its radiation resistance is also weak, and its application in space needs to be verified [12]. Figure 2a presents the structure of a TOPCon silicon solar cell.

IBC technology can be integrated with other technologies. Heterojunction back contact (HBC) has the low-temperature heterojunction passivation characteristics of HJT, and combined with the back contact structure of IBC, there are no front grid lines and back finger electrodes. For space application, the HBC structure has the following unique advantages: the front-side grid-free design reduces optical loss under wide-angle solar illumination in orbit, and the low-temperature heterojunction passivation process can effectively suppress carrier recombination caused by displacement damage under proton irradiation. Wang et al. [13] expounded a new type of silicon solar cell with HBC structure. This structure combines the advantages of TOCon passivation contact and local laser doping technology to solve the problems of complex process and high cost in the production process of traditional IBC silicon solar cells. The back design features a unique cross-finger carrier transport channel, and a silicon-based solar cell with a conversion efficiency of 27.81% and a fill factor of 87.55% is successfully fabricated. The photoelectric conversion efficiency of this solar cell is the highest among global silicon-based solar cells. This work provides an innovative device physics model and process path for designing the next generation of low-cost and high-efficiency back-contact silicon solar cells. TBC combines the passivated contact of “tunnel oxide passivation layer + doped polysilicon” of TOPCon with the IBC back contact structure. It has no front grid lines and back interdigitated electrodes and is a high-efficiency crystalline silicon cell technology in the post-TOPCon era. Tong et al. [14] adopted the TBC technology on a commercial 350.0 cm^2^ single-junction silicon solar cell, obtained an area efficiency of 27.03%, and created a new record. Optimize transparent conductive films (TCFs) and improve metallization processes to help achieve breakthroughs, thereby reducing resistance losses and optical shading. The author elaborately expounds the methods to ensure the uniformity of the nano-film on large-area substrates and solve the edge compounding problem. This achievement is a key milestone for the expansion of the laboratory efficiency of silicon-based solar cells to large-scale production. Hollemann et al. [15], based on the polycrystalline silicon oxide junction (POLO), developed an IBC cell with a conversion efficiency of 26.1%. The core of this research is the detailed and quantitative analysis of the electrical and optical loss mechanisms in the cell. By combining experiments and simulations, the authors found that bulk recombination, Auger recombination, and parasitic absorption in the contact region are the main loss sources. This research at that time created a new efficiency record and pointed out the optimization directions for reducing free carrier absorption and enhancing the breakdown characteristics of the PN junction. IBC and these technologies have good radiation resistance, among which TBC has particularly excellent radiation resistance, which can reach a level close to that of multi junction solar cells. The IBC technology has already undergone technical verification on some small satellites and is a very promising solution. Figure 2d presents the structure of their solar cells.

Although silicon-based solar cells have been replaced by more suitable multi-junction solar cells, it is also important to study their radiation tolerance in order to enable these new silicon-based solar cells to be applied in space. Fedoseyev and Herasimenka used GEANT4 to simulate the radiation effect of silicon solar cells [16]. Yamaguchi et al. [17] conducted research on the radiation tolerance of advanced silicon-based solar cells, discussed non-radiative recombination and resistance loss in silicon space solar cells, analyzed the radiation degradation of silicon space solar cells, and also discussed the effects of substrate carrier concentration and cell thickness on the radiation impedance of silicon space solar cells. However, their research is still limited, only discussing Passivated Emitter Rear Locally diffused (PERL) cells, hetero-junction solar cells and back contact solar cells. The new silicon-based solar cell technology still has great prospects, and more space verification experiments are needed to promote their application on satellites.

The bandgap of silicon-based solar cells is not excellent enough, which limits theoretical efficiency; in addition, their specific power is also relatively low. So, it is necessary to find more efficient solar cells. The efficiency and radiation resistance of III/V solar cells are superior to silicon-based solar cells. They are the most mainstream satellite solar cells.

#### 2.1.2. III-/V-Based Solar Cells

Multi-junction solar cells are currently the most mature (TRL 9) and efficient solar cells [18]. The current mainstream solar cells used on satellites are three-junction GaAs solar cells. The emerging Inverted Metamorphic Multi-Junction (IMM) cells have also been applied in satellites. France et al. [19] respectively obtained efficiency of 39.5% under air mass 1.5 (AM1.5) condition and efficiency of 34.2% under air mass 0 (AM0) condition in the aspect of thick quantum well superlattices of triple-junction cells. The middle layer of this new type of quantum well structure relies on strain to achieve balance, expands the infrared absorption spectrum without lattice defects, and can also enhance current matching. This design uses IMM technology and demonstrates the potential of the quantum well structure in generating current in multi-junction solar cells. Geisz et al. [20] carried out relevant research on six-junction III/V solar cells, and the conversion efficiency reached 47.1% under 143-sun concentration. The band structure of this solar cell is relatively complex. Materials such as AlGaInP, AlGaAs, GaAs, and GaInAs are used to match the wavelength bands of the sunlight spectrum. Researchers use flip-chip growth and a compositionally graded buffer layer technology to control the dislocation density under extreme lattice mismatch conditions. This is one of the highly efficient milestones in the history of photovoltaic technology and photoelectric conversion. However, these two solar cells are currently in the laboratory verification stage. Before they can be applied to space, their radiation tolerance and other indicators need to be verified. Figure 2e presents the structure of a III-/V-based solar cell.

As the most mainstream satellite solar cells today, III-/V-based solar cells have good radiation resistance [21]. Zhao et al. [22] compared the long-term in situ performance of silicon and gallium arsenide solar panels in mid-Earth orbit. The degradation rates of two types of solar panels after 10 years of operation in medium Earth orbit are 13.2% and 9.6%, respectively. This proves that the radiation resistance of III-/V-based solar cells is superior to that of silicon-based solar cells.

The core epitaxial growth, electrode metallization, and stacking/integration processes used in the manufacture of III/V group solar cells are relatively costly. Currently, research on alternative manufacturing methods is carried out. Among the core epitaxial growth processes, molecular beam epitaxy (MBE) and metal–organic chemical vapor deposition (MOCVD) each have their own advantages: atomic-level precision, low defect density, fast growth rate, good large-area uniformity, and mature large-scale production capabilities. The research by Tukiainen et al. [23] shows that the combination of MBE and MOCVD can successfully manufacture GaInP/GaAs/GaInNAs solar cells. Figure 2f presents the corresponding structure. Dilute nitrides (GaInNAs) are used as a base material with an energy of 1.0 eV. Introducing them is important for lattice-matched triple-junction solar cells, although high-quality growth still has certain challenges. In this study, MBE technology is used to pre-control the composition and defects of the dilute nitride layer, thereby greatly improving the quantum efficiency of the base cell. This hybrid growth technology provides a flexible and efficient manufacturing solution for producing multi-junction solar cells with high performance and low defect density. Dai et al. [24] studied the use of low-temperature silver paste screen printing to fabricate electrodes for III/V solar cells, replacing the expensive and complex vapor deposition photolithography process. Focusing on optimizing the rheological properties of the silver paste and the sintering temperature, the contact resistance is reduced, and the aspect ratio of the gate lines is increased without damaging the fragile surface of III/V. The experimental results show that the solar cell performance fabricated by the optimized screen-printing process is similar to that of the traditional method, which lays the foundation for reducing the manufacturing cost and expanding the scope of III/V solar cells in the non-space application market Schygulla et al. [25]. There are improvements in wafer bonding technology, and III/V/Si triple-junction solar cells with III/V/Si at both ends are manufactured. The power conversion efficiency of these cells reaches 36.1% under the AM1.5g standard. The focus of the research is to solve the problem of the trade-off between optical transmission and heat conduction at the bonding interface. After carefully designing the current matching, the series connection of the GaInP/GaAs top cell and the Si bottom cell can achieve large current output. Chen et al. [26] prepared an ultra-thin GaAs solar cell with a silver nanostructure back reflector. Under the 205 nm thick absorption layer, the conversion efficiency reached 19.9%. By designing a specific morphology of the nanostructure back reflector, this study greatly enhanced the light trapping effect of the ultra-thin absorption layer and also used the Fabry–Perot resonance effect to maximize the absorption of photons. This study shows that the advanced photon management structure can ensure the function of high-performance photoelectric conversion and can also reduce the use of III/V materials, thereby reducing the mass and cost of space solar cells.

The main reason for the performance degradation of space solar cells is the irradiation of high-energy particles. When these high-energy particles collide with the material of solar cells, the transmitted energy causes the lattice atoms to shift from their original positions and cause displacement damage, thereby reducing the diffusion length of minority carriers and leading to a decline in the performance of solar cells. Radiation studies on the constituent subcells of InGaP/GaAs/InGaAs inverted metamorphic triple-junction (IMM3J) solar cells have shown that the InGaAs bottom subcell exhibits the most significant degradation under proton and electron irradiation. The reduction in minority-carrier diffusion length leads to a pronounced decrease in short-circuit current, making the InGaAs subcell the primary factor limiting the overall radiation resistance of IMM3J devices. These findings provide important guidance for the development of next-generation radiation-hardened space solar cells [27]. The equivalent flux method and equivalent displacement damage dose (DDD) are the two main methods for evaluating radiation damage in solar cells [28]. The equivalent fluence approach converts irradiation from different particle energies into an equivalent fluence of a reference particle using relative damage factors. The DDD method quantifies lattice damage through the product of particle fluence and non-ionizing energy loss (NIEL). They provide a more physically meaningful and widely accepted framework for assessing radiation effects in modern multi-junction space solar cells.

Although the development of multi-junction solar cells has become very mature, new materials are receiving attention.

#### 2.1.3. Thin-Film Solar Cells

The thin-film solar cells have advantages such as high specific power, flexibility, and low cost. The main materials are CIGS, CdTe, and α–Si. With the rise in microsatellites, the high specific power, flexibility, lightweight and other excellent characteristics of flexible thin-film solar cells make them a promising choice for microsatellites. Ascent Solar Technologies has produced CIGC cells with a specific power of 1900 W/kg at AM0. However, traditional triple-junction GaAs solar cells typically have only 100–300 W/kg, while the new IMM triple junction solar cells can achieve 500–1000 W/kg. However, thin-film solar cells are still a new technology. There are very few in-orbit experiments conducted on them, and most of the achievements are at the laboratory stage. Their radiation tolerance and reliability have not been verified, so they exist merely as a promising solar cell. Figure 2c presents the appearance of a thin-film solar cell.

CIGS is a thin-film solar cell with relatively high efficiency [29]. Tomita et al. [30] developed a cost-effective CIGS solar cell and confirmed its strong radiation resistance. Its excellent performance makes it the best material for thin-film solar cells, and its good radiation resistance also makes it very promising in the aviation field [31]. However, there are drawbacks, such as the use of rare precious metals indium and gallium, and the need for vacuum co-evaporation or sputtering during manufacturing, resulting in relatively high costs. So, people are always researching the manufacturing methods of CIGS. Lontchi et al. [32] optimized the low-temperature manufacturing process of CIGS solar cells on flexible substrates, studied the influence of the composition of the CIGS absorption layer during low-temperature deposition, and also studied replacing copper with silver to improve the solar cell performance, achieving an efficiency of 17%. Although some countries have already installed CIGS solar cells on CubeSats, long-term space qualification of CIGS solar cells remains incomplete, particularly regarding thermal cycling durability, packaging reliability, and deployment-induced mechanical degradation. Figure 2g shows the structure of CIGS cells.

In photovoltaic research, copper zinc tin sulfide (CZTS) is an economical and efficient alternative material to CIGS. Most current research is to improve its low efficiency. For example, Yin et al. [33] obtained a certified efficiency of over 13% through the graded bandgap design in sulfide Kesterite solar cells. The wide bandgap of pure sulfide CZTS material is suitable as the top cell of a tandem, but there is a serious voltage loss. The built-in electric field is constructed by forming a composition gradient at the depth of the absorption layer. This enhances the separation and collection of photogenerated carriers and also reduces the reverse saturation current. This indicates that precise bandgap engineering can achieve efficient photoelectric conversion in selenium-free Kesterite materials.

CdTe (cadmium telluride) is much cheaper than CIGS and has a small difference in efficiency. It was once carried aboard a CubeSat, but its idle application data were quite impressive. It mainly remained in the testing phase in a laboratory environment. Its scalability and repeatability are at the highest level. Zhao et al. [34] developed a single-crystal cadmium telluride solar cell with an open-circuit voltage greater than 1 V and an efficiency of 17%. Traditional polycrystalline cadmium telluride cells are often limited by the open-circuit voltage caused by grain boundary recombination. The author grows high-quality CdTe single crystals on a lattice-matched InSb substrate by molecular beam epitaxy (MBE) and performs in situ As doping to improve high-concentration p-type doping and overcome the comp effect. This achievement shows that CdTe has good photovoltaic potential. Proton irradiation experiments demonstrated that ultralightweight CdS/CdTe thin-film solar cells exhibit promising radiation tolerance for space applications. After exposure to 15 MeV protons with a fluence of 1 × 10^15^ cm^−2^, equivalent to more than 2000 years in low Earth orbit, the cells retained photovoltaic functionality despite a 70% reduction in conversion efficiency. Moreover, the specific power has decreased from 358 W/kg to 109 W/kg. The experiments highlight the potential of CdTe-based thin-film photovoltaics for lightweight and long-duration space missions [35]. However, due to its low efficiency and toxicity, its prospects for application in space are limited.

The radiation resistance of amorphous silicon (α–Si) is better than the other two. It has been tested in orbit on some CubeSat satellites and has served as an auxiliary power source for the satellites. After irradiation, it can partially undergo annealing self-repair and remain flexible, but its efficiency is relatively low. So, improving efficiency has become the focus of research. Kim et al. [36] fabricated a polymer-amorphous silicon (α–Si) hybrid tandem photovoltaic cell with an efficiency of 10%. This device combines the characteristics of high visible light absorption of organic semiconductors with the advantages of amorphous silicon infrared through the optical interconnection layer, thus realizing efficient carrier tunneling. In addition, this study uses photonic crystals to enhance light absorption. This hybrid structure creates a new design for low-cost, solution-processed flexible photovoltaic devices, demonstrating the potential of the organic–inorganic hybrid system.

The efficiency of traditional flexible solar cells is low. Perovskite solar cells have the advantages of high light absorption, adjustable band gap and low cost, so they are preferentially developed as the third-generation solar cells.

#### 2.1.4. Perovskite Solar Cells (PSCs)

PSCs are new solar energy technologies with lower cost, higher mass power density and radiation resistance. They have a unique “self-healing” ability after directional proton irradiation. Experiments show that high-energy protons cause lattice damage and performance degradation, but then defects can be repaired and the solar cell performance can be significantly restored through electron ionization (similar to light or electron injection). Perovskite materials have the unique advantage of the electron ionization-induced repair mechanism (EIIH), and they have excellent survivability and application potential in space applications in complex radiation environments [37]. Figure 2i shows the appearance of perovskite solar cells. Figure 2j shows the schematic diagram of perovskite crystal cell. Figure 2k shows the structure of a kind of perovskite solar cells.

Due to the excellent radiation resistance of PSCs, many studies focus on in situ space environment experiments of perovskite solar cells. Miyazawa et al. [38] place PSC in a low-temperature environment representing the cosmic environment for 8 MeV proton irradiation testing and evaluate its power generation characteristics. Durant et al. [37]’s detailed analysis was conducted on the performance changes in PSC after receiving different types of energy radiation. In addition, there are many studies on the radiation resistance characteristics of PSCs [39,40,41,42].

Multi-junction technology is common on PSCs. All-perovskite tandem solar cells (TSCs) use tunneling junctions to integrate wide/narrow bandgap sub-cells to achieve spectral splitting, thereby improving the photovoltaic conversion efficiency (PCE). Many studies focus on this. Lin et al. [43] developed 3D/3D perovskite heterojunctions, suppressed non-radiative interface recombination and enhanced charge extraction. The all-perovskite tandem solar cells by Wang et al. [44] have a record efficiency of 28.5% (certified as 28.0%). To apply all-perovskite tandem cells on a large scale, scalable manufacturing technologies are needed, and many researchers are working on this. Fu et al. [45] pointed out that the incorporation of piracetam can broaden the bandgap of perovskite, which is used to fabricate high-performance scalable devices. The efficiency of their 1.02 cm^2^ all-perovskite tandem cell reaches 28.2%, and the certified value is 27. When transitioning from small-area devices to large-area devices, the photovoltaic conversion efficiency (PCE) only loses 0.51% (3%). Piracetam shows good performance in perovskite materials, creating a way for scalable and efficient all-perovskite tandem solar cells.

Non-radiative recombination in perovskite solar cells (PSCs) is caused by structural defects. Defect passivation and interface engineering techniques are adopted to solve this problem. Tian et al. [46] constructed a light-stable donor-acceptor interface for inverted perovskite solar cells to reduce energy loss. However, interface non-radiative recombination is still the main factor limiting the efficiency of inverted cells. Modify the interface using a new type of light-stable mol layer to improve the energy level matching and eliminate deep-level defects. This design ensures a high open-circuit voltage and at the same time improves the long-term stability of the device under ultraviolet light, solving the problem of light aging of the inverted structure. Yu et al. [47] treated NiO NPs (HTL) with H_2_O_2_ to make it more able to combine with organic phosphonic acid self-assembled monolayers (SAMs) and inhibit interface recombination. There are devices with a certified PCE of 25%. The efficiency reaches 2%. After 500 h of accelerated aging at 85 °C, it retains 85% of the initial efficiency. Yuan et al. [48] prepared a new SAM molecule MeOF–4SHCz, aiming at the rich Ni^3+^ in the NiO_x_ sub-layer, and achieved the redox reaction at the interface. The overall coverage and uniformity of self-assembled monolayers on NiO_x_ are improved, and the power conversion efficiency (PCE) reaches 26.5%, with the certified value being 26.28%.

Perovskite solar cells (PSCs) have certain potential in tandem, being able to suppress interface recombination and thus reduce energy loss. Their integration with other cells such as silicon, CIGS or organic ones can improve efficiency. This enables multi-junction cells to receive a wider range of sunlight and achieve maximum energy conversion [49]. Liu et al. [50] carried out relevant research on double-terminal monolithic perovskite/silicon tandem solar cells. Use nanoscale LiF ultra-thin layers and depositing diammonium diiodide molecules. Design a double-layer interwoven passivation strategy, combining effective electron extraction and good non-radiative recombination suppression. For this perovskite/silicon tandem cell, the photoelectric conversion efficiency is 33.89%, the fill factor is 83.0%, and the open-circuit voltage is approximately 1.97 V. Li et al. [51] demonstrated a perovskite/perovskite/c-Si triple-junction tandem solar cell, which broadens the light response range by precise bandgap matching. The open-circuit voltage (V_oc_) of this cell is close to 3.0 V, and the photoelectric conversion efficiency (PCE) is 25.0% (certified efficiency: 24.19%), which is the highest certified efficiency of perovskite triple-junction stacked solar cells. The work relies on the paradigm of material design and breaks through the plateau of the performance of perovskite triple-junction stacked cells.

However, as an emerging photovoltaic technology, perovskite solar cells currently remain at a low technology readiness level (TRL 2–4) with limited in-orbit validation, and their performance cannot be fully assessed based solely on laboratory-scale results. Although state-of-the-art perovskite solar cells have demonstrated operational stability exceeding 10,000–20,000 h under laboratory stress tests, their long-term lifetime in space remains uncertain due to the lack of extensive flight heritage and the combined effects of radiation, vacuum, ultraviolet exposure, and thermal cycling, and is still expected to be shorter than that of mature space photovoltaics such as silicon and III–V multi-junction solar cells. Several critical challenges remain unresolved, including outgassing in vacuum environments and instability under thermal cycling conditions. At elevated temperatures, the volatility of organic cations and halide anions in the perovskite lattice is a major concern, leading to structural instability and performance degradation. In addition, the thermal stability of organic charge transport layers (CTLs) plays a critical role in determining device performance under high-temperature conditions. Moreover, the use of metal-based electron transport layers has been reported to introduce increased series resistance at low temperatures (e.g., 180 K), which is attributed to reduced carrier transport efficiency and weakened interfacial charge extraction under cryogenic conditions [52]. Cheacharoen et al. [53] investigated the effect of encapsulation on the thermal cycling stability of PSCs and found that achieving a 25-year operational lifetime requires encapsulation materials with a sufficiently high elastic modulus. Kirmani et al. [54] proposed an encapsulation strategy based on the formation of a SiO_x_ barrier layer via a simple thermal deposition process. Encapsulated PSC devices retained more than 95% of their initial efficiency after exposure to 50 keV protons at a fluence of 1 × 10^15^ p cm^−2^. This high retention is attributed to the ~1 µm-thick SiO_x_ layer, which effectively mitigates proton penetration and suppresses radiation-induced degradation. Further research should focus on improving encapsulation strategies, environmental stability, and long-term reliability under space conditions. Besides encapsulation engineering, device architecture optimization has emerged as another promising approach to enhance perovskite photovoltaic performance and stability. Recently, CsPbI_3_:EuI_2_ perovskites have been successfully integrated into porous gig-lox TiO_2_ frameworks, forming a bilayer structure that combines efficient electron extraction with reduced lattice strain [55]. The porous TiO_2_ scaffold facilitates charge transport and improves the structural adaptability of the perovskite phase while maintaining a lightweight thin-film configuration. Although this architecture was originally developed for semitransparent photovoltaic applications, its enhanced carrier extraction characteristics and reduced material consumption may provide valuable insights for the development of future lightweight space photovoltaic systems. Nevertheless, dedicated investigations under vacuum, radiation, atomic oxygen, and thermal-cycling conditions are still required to evaluate the suitability of such architectures for long-duration space missions.

Figure 2 presents the complete technical system of space photovoltaic cells as a whole, which is the most dominant primary energy supply technology for satellites at present. The figures cover structural design, fabrication processes and performance features of multiple types of space-grade photovoltaic devices, including crystalline silicon cells, III-V multi-junction cells, CIGS thin-film cells and perovskite tandem cells, to match different satellite mission demands as follows: high-power satellites adopt high-efficiency III-V multi-junction cells; micro-/nanoflexible satellites employ lightweight thin-film or perovskite cells; low-cost low Earth orbit (LEO) satellites use mature crystalline silicon cells.

It intuitively reflects the technical trade-offs of space photovoltaic technology in photoelectric conversion efficiency, weight, radiation resistance and cost, and forms the full technical panorama of the “direct light-to-electricity conversion” main route for satellite energy harvesting.

**Figure 3:**
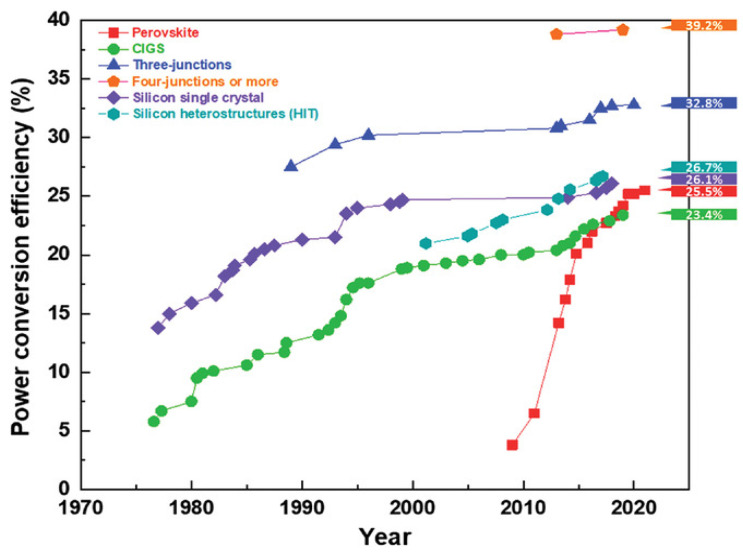
Composition of the PCE of solar cells with different materials [62].

Figure 3 shows the composition of the PCE of solar cells with different materials. The plot illustrates the steady advancement of solar cell technologies over five decades, with distinct performance trajectories observed for different material systems. Multi-junction compound semiconductor cells demonstrate the highest efficiencies, reaching 39.2% for four-junction or more configurations and 32.8% for three-junction devices. Silicon-based technologies, including monocrystalline silicon and silicon heterojunction (HJT) cells, have shown consistent incremental improvements, achieving record efficiencies of 26.1% and 26.7%, respectively. Copper indium gallium selenide (CIGS) thin-film solar cells have also progressed steadily, attaining a maximum efficiency of 23.4%. Notably, perovskite solar cells exhibit an unprecedentedly rapid efficiency increase since their emergence around 2010, surging from below 5% to 25.5% within a decade, making them the fastest-growing photovoltaic technology in terms of efficiency improvement.

Table 1 presents the performance and related characteristics of various types of solar cells. The table shows that III/V solar cells have great advantages in energy conversion efficiency. It can make full use of solar energy by adjusting the energy band. The perovskite solar cell can optimize its performance in different aspects by stacking and connecting with other materials in series. Its low cost has great potential for development. The energy conversion efficiency of silicon-based and thin-film solar cells is average, but the technology of silicon-based solar cells is relatively mature, and many new technologies have been extended, which is very close to the theoretical limit of silicon-based power generation efficiency. Thin film solar cells can also be used in the aerospace field because of their high mass-specific power and ultra-thin characteristics.

**Table 1:** Comparison of solar photovoltaic power generation.

Material	Year	PCE	Aperture-Area	FF	Characteristic	References
Six-junction III–V	2020	39.2% (AM1.5g)	0.25 cm^2^	83.5%	Precise control of subband gaps to achieve maximum AM0 full-spectrum absorption	[20]
GaInP, GaInAs/GaAsP, GaInAs	2022	34.2% (AM0)	0.25 cm^2^	-	The band gap of the intermediate cell is modified by using a thick GaInAs/GaAsP strain-balanced quantum well (QW) solar cell with excellent absorption and voltage.	[19]
Perovskite	2022	19.1% (AM1.5g)	6.25 cm^2^	94.7%	Laser stripped all perovskite series modules processed by scalable manufacturing methods (blade coating and vacuum deposition).	[63]
(PbI2) 2RbCl Perovskite (flexible)	2022	25.6% (AM1.5g)	0.108 cm^2^	82.7%	The perovskite phase is stabilized and the band gap is reduced by the introduction of the passive non-active phase (PbI2)2RbCl.	[64]
Perovskite/CIGS (flexible)	2022	24.2% (AM1.5g)	1.04 cm^2^	71.2%	Perovskite and CIGS combination.	[65]
SHJ	2023	25.94% (AM1.5g)	274.4 cm^2^	85.71%	Amorphous undoped SnO_x_ TE with copper electrodes instead of indium-based TE with silver electrodes.	[9]
IBC	2025	27.81% (AM1.5g)	166.1 cm^2^	87.55%	A hybrid fingertip back-contact solar cell was developed by combining advanced full-surface passivation technology with laser-tunneling contact.	[13]
GaInP/GaInAsP//Si	2025	36.1% (AM1.5g)	3.987 cm^2^	86.0%	The back heterojunction is realized, the middle cell is improved, and the advanced metal dielectric back-side grating is used to enhance the light capture of the silicon bottom cell.	[25]
Perovskite/silicon (flexible)	2026	33.6% (AM1.5g)	1 cm^2^	81.9%	Realization of reactive plasma deposition (RPD) needle and indium oxide co-doped with hydrogen (ICO:H) composite layer	[66]

### 2.2. Electromagnetic RF Power Generation

The universe has a rich source of electromagnetic waves. In addition to visible light, many electromagnetic wave bands can be used for satellites to receive energy. These sources include celestial bodies such as stars, supernovae, cosmic background radiation, and electromagnetic waves generated by human activities. However, the output power of radio frequency (RF) energy harvesting in space is only nW to μW, which greatly limits its applications on satellites. It can only supply power to low-power loads onboard, such as sensors for satellite platform health monitoring, wireless network nodes and wake-up circuits. The usual working mode of this system is to collect RF energy to charge the battery, and then when the power is sufficient, wake up the low-power load to work. After the work is completed, it will sleep again and cycle continuously. Directed microwave power transmission (MWPT) is a wireless energy transfer technology that converts electrical energy into highly directional microwave beams and delivers it over long distances to a receiving rectenna for RF-to-DC conversion. Compared with ambient RF energy harvesting, MWPT can provide significantly higher power density and more controllable energy delivery. Therefore, it has been considered a promising approach for future space-based solar power systems, satellite-to-satellite energy transfer, and auxiliary power supply for spacecraft.

The mature electromagnetic energy harvesting technology for terrestrial Internet of Things (IoT) can be migrated to satellite platforms in terms of core principles, including rectifier topologies, impedance matching circuits and low-power power management. Since the power demand of ultra-low-power devices is similar in both scenarios, this technology is applicable to power micro-sensors, wireless sensor nodes, wake-up circuits and data retention circuits on satellites. However, there are obvious differences in operating environments and power limits. Ambient electromagnetic radiation in space only provides nanowatt to microwatt power, and directed microwave wireless power transfer can merely reach 10–100 W. Accordingly, the terrestrial schemes designed for high-power loads and main power supply cannot be adopted for satellites. In addition, antennas, protection and heat dissipation structures optimized for near-ground atmospheric environments must be redesigned to adapt to space vacuum, extreme temperature variation and cosmic radiation.

Broadband and high-gain antenna arrays are essential for harvesting electromagnetic energy from RF sources such as MWPT systems. Ha et al. [67] develop a low-profile, wide-angle, bandwidth-enhanced rectenna. The proposed rectification antenna can maintain robustness to changes in received power and incident angle (from −65° to 65°), while allowing broadband operation from 11.5 GHz to 12.5 GHz. The proposed rectifier antenna has a power density of 2.86 mW/cm^2^ and an almost constant output voltage over a wide range of incident angles and operating frequencies, with an output voltage of 1.88 V and an RF to DC power efficiency of 45% Das et al. [68]. There is a high-efficiency wideband hexagonal rectenna array, whose frequency band is between 3.6 and 6.45 GHz, with 360° omnidirectional coverage and high gain characteristics. It is very important for low-orbit satellites with changing directions. It is necessary to ensure that energy can be stably received regardless of its position with ground stations or inter-satellite links. Under a 1 kΩ load resistance and an input power of 9 dBm, the proposed rectenna achieved a maximum power conversion efficiency of 59.82% and an output voltage of 1.9 V at 5.8 GHz. Moreover, efficiencies above 40% and output voltages exceeding 1.3 V were maintained across the 3.6–6.45 GHz frequency range, demonstrating its potential for broadband low-power wireless energy harvesting applications. Nosrati et al. [69] proposed a wide-band rectenna based on a hybrid “sandwich” power divider. The sandwich rectenna operates with an efficiency of over 28% in the frequency range of 2 to 18 GHz, with input power ranging from −15 to +15 dBm. The system functions effectively even at very low radiation power densities. This is very suitable for space environment.

Satellite electromagnetic energy harvesting is used to power low-power components, and the design of low-power rectifier circuits is important. Due to limited space, the circuit needs to be compact. Assimonis et al. [70] proposed an electromagnetic rectenna design without a matching network suitable for micro-watt input power. Traditional impedance matching networks often introduce additional insertion loss, and the efficiency will be greatly reduced at low power. In this study, the conjugate matching of the high inductance of the antenna and the capacitance of the rectifier diode is used to simplify the circuit, improve the sensitivity and conversion efficiency of the system in the low-power state, and thus provide a compact power supply solution for the mini-satellite components with limited space. Parween et al. [71] proposed a dual-band dual-polarized rectenna system, which is applied to energy harvesting at 3.6 GHz and 5 GHz.8 GHz. Optimize the impedance matching rectifier circuit to extend the working range of the system at low input to −20 to 20 dBm, confirming that it is feasible to continuously power low-power circuit in complex electromagnetic environments.

Rectennas are the most mature RF energy harvesting technology and represent the most readily transferable solution for space applications. The fundamental architecture, including receiving antennas, impedance matching networks, RF–DC rectifiers, and power management circuits, can be directly adopted from terrestrial systems. However, several components require redesign for orbital deployment. Semiconductor rectifiers, particularly Schottky diodes, may suffer from radiation-induced degradation and parameter drift under high-energy particle exposure. In addition, thermal cycling between sunlight and eclipse conditions can alter impedance matching characteristics and reduce conversion efficiency. Structural designs must also accommodate launch vibration, vacuum compatibility, and deployment constraints. Therefore, while the operating principle remains unchanged, component-level space qualification is required before implementation.

**Figure 4:**
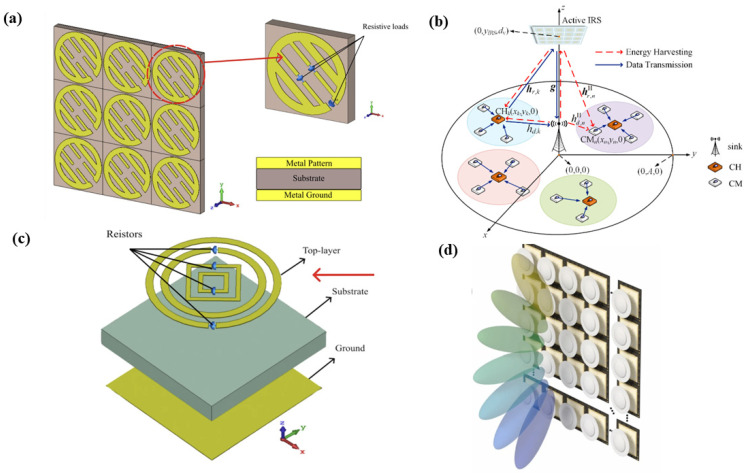
Different electromagnetic energy harvesting systems. (**a**) Metamaterial array structure diagram [72]. (**b**) Schematic diagram of IRS-assisted system [76]. (**c**) Metamaterial structure diagram [74]. (**d**) Lens-enhanced electromagnetic energy harvesting system [77].

New materials and smart reflectors are changing contemporary electromagnetic energy harvesting. Metamaterials have the ability to absorb electromagnetic waves, broadband characteristics, and polarization insensitivity, and have broad prospects in electromagnetic energy harvesting. Ullah et al. [72] developed a compact dual-frequency energy harvester based on double-negative metamaterials. With the unique electromagnetic properties of metamaterials (negative permittivity and negative permeability), this device realizes efficient resonance in the ISM band in a very small size. Two types of metamaterials suitable for different frequency bands are also developed [73,74]. Figure 4a,c shows the structure of the meta-array. The miniaturized design saves space and maintains a high absorption cross-section, which can be applied to receive leaked radio frequency energy in the high-density electromagnetic environment of satellites. Wang and Yu [75] studied the radio frequency energy harvesting method using intelligent reflecting surface (IRS) in cognitive radio sensor networks. Relying on the active reconstruction of the wireless channel by IRS, the system can focus the radio frequency signal to the sensor node and has limited energy capture capability. In complex satellite environments or constellation networks, this technology reduces signal occlusion and multipath fading and improves the reliable efficiency of the wireless charging link. Wang and Zhu [76] integrated IRS to support downlink EH and uplink trans, so that the lifetime of EH CRSN network is extended. They constructed a constrained non-convex optimization problem to maximize the net energy gain. Figure 4b shows the schematic diagram of the IRS-assisted system. In addition, Joshi et al. [77] used lenses to enhance the collection of electromagnetic waves. Figure 4d shows the structure of its system.

Regarding these promising technologies, certain issues need to be addressed. Metamaterial-based RF harvesters can partially benefit space applications through their electromagnetic field localization and resonant absorption mechanisms, which are independent of the operating environment and can be directly transferred. However, the dielectric substrates and metallic resonators used in most terrestrial designs are not optimized for prolonged exposure to ionizing radiation, vacuum conditions, and severe thermal cycling. Radiation-induced changes in dielectric properties and thermally induced dimensional variations may shift resonance frequencies and reduce absorption efficiency, necessitating material redesign and environmental qualification. Intelligent reflecting surfaces (IRSs) can theoretically enhance RF energy concentration through programmable phase control, and the wavefront manipulation principle itself remains applicable in space. Nevertheless, current IRS architectures depend on large numbers of tunable unit cells, semiconductor phase shifters, biasing networks, and real-time control circuits, which are susceptible to radiation-induced degradation and introduce additional power consumption and reliability concerns. Moreover, maintaining precise phase control across large deployable structures presents significant engineering challenges for spacecraft implementation. Lens-enhanced RF harvesting systems retain their ability to focus incident electromagnetic waves and increase received power density, making the antenna–lens integration concept transferable. In contrast, conventional dielectric lens structures are often bulky and optimized for terrestrial operation; their mass, thermal deformation under extreme temperature fluctuations, and compatibility with launch and deployment requirements limit direct adoption in spacecraft systems, requiring substantial structural and material modifications.

Given the crucial role of solar energy in satellite energy harvesting, many researchers combine radio frequency (RF) with solar energy systems. Bito et al. [78] developed a new electromagnetic–solar energy harvesting power communication system that operates in the 2.4 GHz ISM band. This system can enable the low-power management circuit in wireless sensors to run by itself and shows good cold-start ability. When the PMU operates independently during cold start or hot start, it keeps the radio frequency input power between −12.6 dBm and −15.6 dBm. Wan et al. [79] encapsulated the solar cell into a mesh patch and made it into a substrate between rectifier circuits. Based on the particle swarm optimization algorithm, a grid pattern with high light transmittance (96.2%) is placed on the surface of the solar cell. Under the condition of the optimal load of 400 Ω, the DC conversion efficiency of the hybrid solar rectenna reaches 96.8%.

Table 2 presents the performance and characteristics of different rectenna antennas. The table shows that the overall efficiency of electromagnetic energy collection is not high, and it is mostly used for low-power circuit power supply. Broadband antenna or antenna array is mostly used for electromagnetic energy collection in satellites, and the energy conversion efficiency of antenna array is higher than that of other antennas. Although the broadband antenna is not the most efficient, it can collect weak electromagnetic waves in the cosmic environment.

Figure 4 demonstrates the technical system of RF wireless energy harvesting and transmission, which is an emerging solution for supplementary energy supply and space energy relaying for satellites. The figures display core components including rectenna units, beam regulation via intelligent reflecting surfaces (IRSs), and integrated energy/data transmission systems for satellite clusters, corresponding to diverse space applications as follows: ground stations or space solar power stations deliver wireless energy to satellites via RF beams; wireless energy relaying among satellite constellations; micro-/nano satellites harvest supplementary energy from ambient RF signals. Unlike photovoltaic technology which relies on direct sunlight, RF energy harvesting supports long-distance, non-line-of-sight energy transmission, and can solve problems such as power supply for satellites in shadowed areas and lightweight energy supply for micro-satellites. It serves as a critical supplementary technical route beyond the primary space energy source.

### 2.3. Thermoelectric Power Generation

Thermoelectric generators (TEGs) have attracted increasing attention as a supplementary energy-harvesting technology for spacecraft because they can directly convert temperature differences into electrical energy through the Seebeck effect. Owing to their solid-state structure, lack of moving parts, high reliability, and long operational lifetime, TEGs are particularly attractive for harsh space environments. Spacecraft are frequently exposed to temperature fluctuations caused by solar illumination, eclipse transitions, equipment operation, and waste-heat generation, providing opportunities for thermoelectric energy harvesting. Although the available temperature gradients on most spacecraft are typically limited and the generated power is generally much lower than that of photovoltaic systems, TEGs can still provide auxiliary power for low-power electronics, distributed sensors, and structural health monitoring systems [82].

Optimizing the dimensionless ZT value of materials is the key to improving thermoelectric conversion efficiency. In recent years, research has expanded from traditional PbTe to systems based on GeTe, skutterudites, and high-entropy alloys.

**Figure 5:**
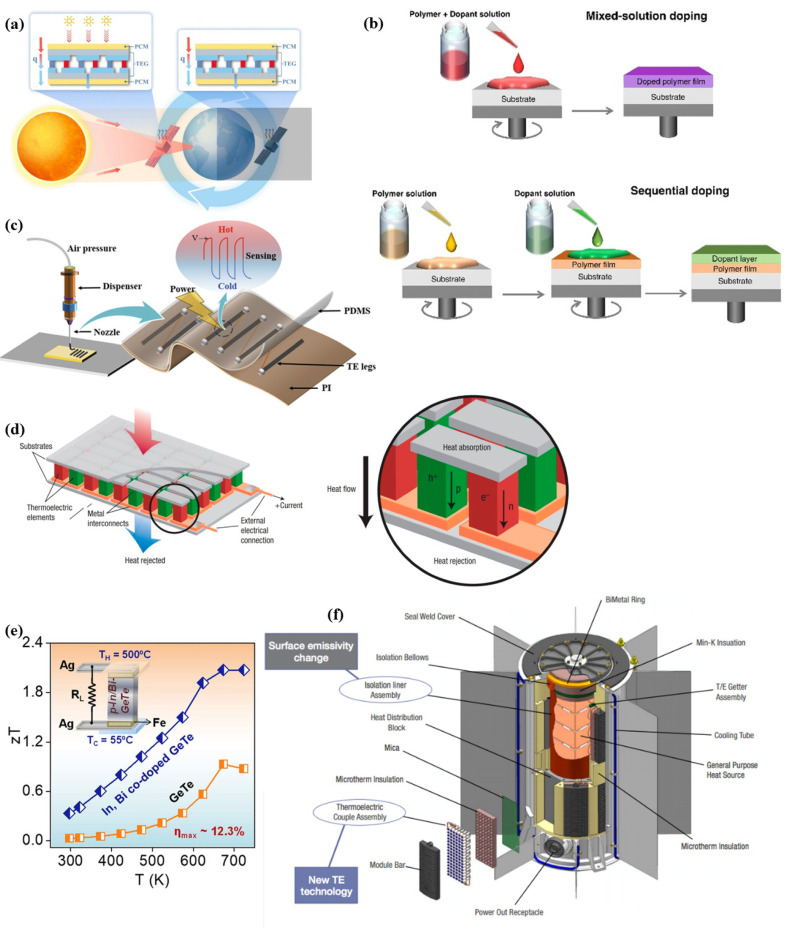
Different thermoelectric energy harvesting systems. (**a**) Phase-change material-based geostationary satellite thermoelectric power generation system [94]. (**b**) Preparation of conjugated polymer thermoelectric material [93]. (**c**) 3D-printed Bi2Te3-based thermoelectric generators [103]. (**d**) Schematic diagram of hot spot generator [82]. (**e**) Properties of Bi-doped GeTe thermoelectric material [83]. (**f**) Enhanced multi-mission radioisotope thermoelectric generator (courtesy of NASA).

GeTe-based materials have attracted attention. High thermal conductivity and low Seebeck coefficient limit the figure of merit ZT of GeTe materials. Perumal et al. [83] used Bi-In co-doping to optimize the carrier concentration of GeTe and reduce the lattice thermal conductivity and obtained ZT~2. The efficiency is about 12.3% at 723 K (and 445 K). Figure 5e shows the properties of bismuth-doped germanium telluride-based thermoelectric materials. Jiang et al. [84] adopted the high-entropy stabilization strategy: adding more cations to the germanium telluride based materials to stabilize the phase with excellent thermoelectric properties. Cationic disorder results in low thermal conductivity of high-entropy materials, but the symmetry is still relatively good and the electrical performance is well-maintained. Multielement alloying suppresses phonon transport by introducing strong lattice distortion, and a conversion efficiency of 13.3% is obtained in GeTe-based materials. Xing et al. [85] used Mo-Al alloy containing 30% high CTE Al as the interface barrier layer material, and optimized the barrier layer composition to develop an efficient GeTe-based module. The efficiency of this component is 7.8% at 500 K, and the power density is 1.1 W·cm^−2^. PbTe is a traditional material. Jood et al. [86] used germanium-induced nanostructures and over-doping to improve the full-band phonon scattering and increased the efficiency of the PbTe component to 12% at 509 K.

The skutterudite is used in the warm area. The cost of skutterudite is relatively high. Tang et al. [87] reduced the cost through solubility design and then improved the performance of Ce–CoSb_3_ skutterudite. They found that the limit temperature dependence of the filling factor is relatively strong, and after optimization, the ZT value of the felt reaches 1.3 at 850 K. Li et al. [88] drew the phase diagram of R-Fe-Sb (R = Ce, Nd, Pr) at 823 K to solve the ambiguous problem of the filling fraction limit in R_x_Fe_4_Sb_12_. High filling fraction makes the structure stable and optimizes the carrier concentration. The peak ZT of Ce_0.98_Fe_4_Sb_12_ is about 1. The average ZT value at 773 K is about 0.65 (in the range of 300–773 K). The role of phase diagram engineering in materials design is a highlight of the research, laying a thermodynamic foundation for p-type rutile-based materials. Zhang et al. [89] found that the lack of reliable diffusion barriers at the electrical interface hinders the application of pyrochlore cobaltate-based thermoelectric (TE) devices in high-temperature waste heat recovery. To solve this problem, interlayer design first attaches importance to low yield strength. The high-entropy method is used to overcome the problem of performance trade-offs in traditional alloys. After 1800 h of aging treatment, the efficiency loss is about 0.5% under a temperature difference of 575 K.

The skutterudite based on Bi_2_Te_3_ is the core material in low temperature and the key to low-grade heat energy harvesting. Figure 5c shows 3D-printed Bi_2_Te_3_-based thermoelectric generators. Zhang et al. [90] doped (Cd, S) at the Sb/Te sites to simultaneously improve the thermoelectric and mechanical properties of Bi_2_Te_3_-based materials. The ZT value of the experimental sample is about 1.5 at 350 K, and the average ZT value is 1. Between 300 K and 500 K, the efficiency is 6.8% under a temperature gradient of 200 K. (Bi, Sb)_2_(Te, Se)_3_ shows the best performance at medium and low temperatures.

Half-Heusler (HH) alloys belong to high-temperature materials. Xing et al. [91] carried out reverse engineering from device to material design, constructed thermal conductivity scaling laws for p-type/n-type materials, optimized module topology, and minimized interface resistance to develop a high-performance thermoelectric device model. The study adopted the n-type Zr_0.5_Hf_0.5_NiSn_0.97_Sb_0.03_ half-Heusler alloy that has a good power factor but a relatively low ZT, and there is also the p-type Nb_0.86_Hf_0.14_FeSb alloy. Eight temp TE modules achieve an efficiency of 10.5% under a temperature difference of 680 K and have a power of 3.1 W/cm^2^, setting two new performance indicators for single-stage TE modules.

Researchers are looking for non-traditional materials for spacecraft. Kilinc et al. [92] prepared Ca_2.7_Ag_0.3_Co_4_O_9_ oxide, and its power factor is 0.50 mW/m·K^2^. This oxide is not as efficient as alloys, but it can remain stable in high-temperature oxidizing environments and can be used in the aerospace field. Wang et al. [93] reviewed the progress of organic thermoelectric materials, pointing out that they have flexibility and low density, which are relatively suitable for powering distributed low-power sensors on satellites. Figure 5b presents the conjugated polymer thermoelectric materials prepared by them. TEGs integrated with PCM are used for spacecraft power supply in environments with extreme temperature changes. Song et al. [94] developed a PCM-based thermoelectric system. It uses the latent heat of PCM to continuously release heat on the shadow side to ensure power supply and regulate the satellite thermal environment. A spacecraft heat energy harvesting device using gallium as PCM has also been developed [95]. These technologies are important foundations for improving the efficiency of thermoelectric power generation. Figure 5a shows the PCM-based thermoelectric power generation system of a geosynchronous satellite.

Despite their advantages of solid-state operation, high reliability, and long service life, thermoelectric generators (TEGs) still face several challenges in practical spacecraft applications. First, the temperature gradients available on most spacecraft are often limited because thermal control systems, insulation structures, and heat conduction paths tend to reduce temperature differences across the TEG modules. As a result, the actual conversion efficiency and power output are typically much lower than those reported under laboratory conditions with large temperature differences. Second, the amount of recoverable waste heat on many satellite platforms, particularly small satellites and CubeSats, is relatively limited, restricting the achievable output power to the milliwatt level in many cases. In addition, thermal contact resistance, packaging losses, and integration constraints can further degrade system performance. Long-term exposure to thermal cycling, radiation, and vacuum environments may also affect the reliability and stability of thermoelectric materials and interfaces. Lukowicz et al. [96] studied the characteristics of TEG under extreme conditions. Therefore, although TEGs are promising as supplementary energy sources and waste-heat recovery devices, their practical implementation in spacecraft is not yet mature (TRL3–5); it requires careful consideration of thermal management, structural integration, and long-term environmental durability.

### 2.4. Radioisotope Thermoelectric Generators (RTGs)

Radioisotope thermoelectric generators (RTGs) are autonomous power systems that convert the heat released by radioactive isotope decay into electricity through thermoelectric conversion. With typical power outputs ranging from tens to hundreds of watts and operational lifetimes exceeding several decades, RTGs provide reliable and continuous power in environments where solar energy is insufficient. Since the 1960s, RTGs have been widely deployed in deep-space exploration missions, including Voyager, Cassini, Curiosity, and Perseverance, becoming one of the most important power technologies for long-duration space missions. Du et al. [97] constructed a physical model for a ~(90)Sr isotope thermoelectric power source. The maximum output power of their isotope energy device is 63.6 We, and the thermoelectric conversion efficiency is 7.6%, but after 15 years, its maximum output power drops to 24.4 We. RTGs have a long operating life and become an ideal power source for deep space exploration vehicles.

Multi-Mission RTGs are currently the mainstream RTGs used for spacecraft. Barklay et al. [98] have proposed integrating a secondary Bi_2_Te_3_ thermoelectric circuit into the MMRTG architecture to recover residual waste heat. The approach can increase both beginning-of-life and end-of-life electrical power by approximately 20% (up to 142 We), although the associated mass penalty significantly reduces the specific power of the system (from 2.37 to 1.68 We/kg). This highlights the trade-off between energy conversion efficiency and mass optimization in next-generation RTG design. Matthes et al. [99] analyzed 21 potential thermocouple configurations to maximize converter efficiency and power density by considering various high-performance high-temperature TE materials and segmentation techniques. Figure 5f shows the structure of enhanced multi-mission radioisotope thermoelectric generator.

Despite their high reliability and decades-long operational capability, MMRTGs are subject to gradual performance degradation during long-duration space missions. In addition to the natural decay of radioisotope fuel, thermoelectric material aging and interface degradation can further affect power generation efficiency. Long-term telemetry data from the Curiosity rover have provided valuable insights into the in-orbit performance degradation of MMRTGs. Analysis of more than ten years of operational data indicates that, although thermoelectric material aging and interface degradation gradually reduce power output, the overall impact on system performance remains limited. The projected end-of-design-life (EODL) power is approximately 73 We, only about 2.2 We lower than previous estimates. Furthermore, performance extrapolation suggests that the MMRTG could continue operating for more than 20 years while still maintaining roughly half of its initial electrical output. These results demonstrate the remarkable long-term stability and reliability of RTG technology, highlighting its suitability for extended deep-space exploration missions where continuous and maintenance-free power generation is essential [100].

Although MMRTGs have demonstrated outstanding reliability and long operational lifetimes in deep-space missions, conventional RTG technology still faces several inherent limitations. The thermoelectric conversion efficiency of current RTGs is typically below 10%, resulting in substantial waste heat and relatively low specific power. In addition, the limited availability and high production cost of Pu-238 have become significant constraints on the deployment of future radioisotope power systems. To address these challenges, increasing attention has been directed toward dynamic radioisotope power systems (DRPSs), which utilize heat engines such as Stirling, Brayton, or Rankine converters to transform radioisotope heat into electricity. Compared with conventional RTGs, these systems can achieve significantly higher conversion efficiencies and reduce radioisotope fuel consumption, making them promising candidates for next-generation deep-space power applications. VanderVeer et al. [101] integrate Stirling convertors into a generator. System modeling shows that their designed DRPS can generate a power of 237 We, a specific power of 3.0 We/kg, and an average degradation rate of 0.9% per year. In response to the shortage of Pu-238 supply, an Americium fueled radioisotope Stirling generator was designed that uses Am-241 as fuel and utilizes Free Piston Stirling Convertors (FPSC) to improve its efficiency [102]. It can output 216 We of power, with a specific power of 1.97 We/kg. However, this radio isotope stirring generator is still in the research stage, and the thermal power density of Am-241 is much lower than that of Pu-238. At present, it can only serve as a substitute for Pu-238.

Beyond technical performance, RTG deployment is severely constrained by critical programmatic and regulatory barriers. First, nuclear launch licensing requires rigorous multi-year safety assessments of accident scenarios, adding significant schedule and cost complexity. Second, export control restrictions on special nuclear material Pu-238 limit RTG production capability to only a handful of nations. Finally, prohibitive costs (a single MMRTG costs ~$100–150 million) restrict their use to only the highest-priority deep space missions. These factors collectively explain why RTGs remain a niche technology despite their unmatched reliability for long-duration missions far from the Sun.

Table 3 presents the performance and characteristics of different thermoelectric generators. The table shows that the power generation efficiency of thermoelectric generators is limited and the difference is small, and the mainstream materials are mainly suitable for different temperature zones. Its application scenarios are mainly deep space and other places where it is difficult to obtain energy. RTG has been applied in deep space detectors with its excellent reliability.

Figure 5 presents the technical system of thermoelectric energy conversion as a whole, which is a key technology for satellite waste heat recovery and deep space exploration energy supply. The figures cover working principles of space thermoelectric systems, device structures, thermoelectric material performance, flexible fabrication processes and space-grade complete machine structures, corresponding to the following two core satellite applications: first, recovering waste heat from on-board electronic equipment and utilizing the temperature difference between the sunlit and shaded sides of satellites for auxiliary power generation, to realize the integration of thermal control and energy recovery; second, acting as the primary energy supply solution (such as radioisotope thermoelectric generators, RTGs) for deep space exploration scenarios far from the Sun with insufficient illumination, providing continuous and stable power output in light-free environments. Compared with photovoltaic and RF technologies, thermoelectric conversion features no moving parts, ultra-high reliability and independence from external light or RF sources, making it the core energy technical support for long-life deep space satellites and satellites operating in extreme environments.

**Table 3:** Comparison of thermoelectric power generation.

Material	Year	η	T_cold_ T_hot_	ZT	Power Density	Characteristic	References
PbTe	2018	12.0%	T_cold_ = 283 K T_hot_ = 873 K (ΔT = 590 K)	1.9	~1.0 W/cm^2^	The lattice thermal conductivity is significantly reduced by the excess doping and nano-phase precipitation, and the high efficiency is achieved.	[86]
n-type Zr_0.5_Hf_0.5_NiSn_0.97_Sb_0.03_ p-type Nb_0.86_Hf_0.14_FeSb	2020	10.5%	T_cold_ = 366 K T_hot_ = 1046 (ΔT = 680 K)	-	3.1 W/cm^2^	Based on the thermal matching criteria and power factor priority principle, the design integrates high conversion efficiency and ultra-high-power density into a single module, rather than solely pursuing the highest ZT value.	[91]
GeTe	2021	7.8%	T_cold_ = 300 K T_hot_ = 800 K (ΔT = 500 K)	2.0	1.1 W/cm^2^	The typical GeTe-based module performance was enhanced by selecting barrier layer materials to improve stability.	[85]
GeTe	2022	13.3%	T_cold_ = 800 K T_hot_ = 294 K (ΔT = 506 K)	2.7 (750 k)	-	The high-entropy effect was utilized to optimize electron and phonon transport, setting a new efficiency record for similar materials.	[84]
Mg2Sn-GeTe	2025	9.0%	- (ΔT = 418 K)	1.4	0.7 W/cm^2^	The efficiency limitation of 2Sn caused by high thermal conductivity is resolved, effectively reducing thermal conductivity while maintaining power factor.	[104]
MMRTG	2023	6.3%	- (2000 Wth)	-	115.3 We 2.7 W/kg	RTG can function for a long time in harsh environments, and its technology is very mature.	[100]

## 3. Spacecraft Electrical Power System (EPS)

### 3.1. Satellite Energy Storage Unit

#### 3.1.1. Ni-H_2_ Batteries

Owing to their excellent cycle life, high reliability, and strong tolerance to harsh space environments, Ni-H_2_ batteries have been extensively used in long-duration satellite and space station missions. These batteries were developed in the 1970s and later applied by NASA to famous spacecraft such as the Hubble Telescope. Energy harvesting in the universe is intermittent, so batteries need to withstand a large number of cycles, in which case other batteries typically have a very short lifespan. Ni-H_2_ batteries have demonstrated operational lifetimes exceeding 15 years with more than 60,000 partial depth-of-discharge cycles [105]. This made them the most successful energy storage technology for spacecraft at that time. However, due to their low energy density (40~75 Wh/kg) and high cost, they were gradually replaced by later Li-ion batteries.

#### 3.1.2. Li-Ion Batteries

Lithium-ion (Li-ion) batteries are currently the dominant energy storage technology in modern spacecraft due to their high energy density, low self-discharge rate, and long cycle life. Li-ion batteries store and release electrical energy through the reversible migration of lithium ions between the cathode and anode during charging and discharging processes. Current commercial Li-ion energy cells typically provide 150–270 Wh/kg and an average voltage of 3.6 V. Compared with traditional Ni-H_2_ batteries, Li-ion batteries provide significantly higher specific energy while reducing system mass, making them particularly attractive for satellite power systems.

As the most popular spacecraft batteries at present, it is important to verify whether they can maintain operation in extreme cosmic environments. Recent studies have demonstrated the feasibility of Li-ion batteries operating under extreme low-temperature conditions relevant to lunar and deep space missions. Using a dedicated testing platform capable of reaching −175 °C, Li_4_Ti_5_O_12_ (LTO)-based cells maintained reversible electrochemical activity even at −100 °C, although significant capacity degradation was observed with decreasing temperature (from 159.04 mAh/g at 25 °C to 7.12 mAh/g at 100 °C) [106]. These results highlight the potential of advanced Li-ion battery technologies for future space missions operating in harsh thermal environments. However, the performance and capacity of Li-ion batteries are highly sensitive to temperature. To maintain optimal operation within the typical temperature range of 20–40 °C, spacecraft generally employ thermal management systems to regulate battery temperature and ensure long-term reliability. Zhang et al. [107] have proposed hybrid thermal-control architectures combining liquid cooling, thermoelectric coolers (TECs), and flat plate heat pipes (FPHPs). Current thermal management research mainly focuses on ground energy storage, with less research on space, and further research is needed.

#### 3.1.3. Next-Generation Batteries

To satisfy the increasing energy demands of future satellites and deep space missions, several next-generation battery technologies are being actively investigated. Solid-state batteries (SSBs) replace flammable liquid electrolytes with solid electrolytes, significantly improving safety and thermal stability while enabling the use of lithium-metal anodes. Recent prototypes have achieved specific energies exceeding 300 Wh/kg, compared with 150–250 Wh/kg for current space-qualified Li-ion batteries. However, high interfacial resistance and limited ionic conductivity at low temperatures continue to restrict their rate capability and long-term cycling performance. Lithium-sulfur (Li-S) batteries have attracted considerable attention due to their theoretical specific energy of approximately 2600 Wh/kg and practical targets of 400–600 Wh/kg, potentially doubling the energy density of conventional Li-ion systems. Nevertheless, their widespread deployment remains hindered by the polysulfide shuttle effect, which often results in rapid capacity fading and cycle lives typically below 500–1000 cycles. Lithium-air (Li-O_2_) batteries exhibit an even higher theoretical specific energy of about 3500 Wh/kg, approaching that of hydrocarbon fuels. Despite this advantage, severe oxygen-electrode degradation, large charge–discharge overpotentials, and poor cycling stability currently limit most laboratory cells to only several tens or hundreds of cycles. Therefore, although these advanced battery technologies offer substantial potential for reducing spacecraft mass and increasing energy-storage capability, significant improvements in durability, reliability, and environmental adaptability are still required before their adoption in practical space missions.

### 3.2. Power Management and Distribution

Power Management and Distribution (PMAD) serves as the central control unit of spacecraft electrical power systems, responsible for collecting, conditioning, distributing, and regulating electrical energy from multiple power sources. In modern satellite power architectures, PMAD enables the coordinated operation of photovoltaic arrays, radioisotope power systems, other energy harvesters, and battery storage units, ensuring stable and reliable power delivery to onboard subsystems and payloads.

The PMAD module is typically composed of Power Conditioning Unit (PCU), Battery Management System (BMS), Power Distribution Unit (PDU), Primary/Secondary Bus Converter, Telemetry and Protection Circuit.

1. Power Conditioning Unit (PCU): The Power Conditioning Unit is responsible for power conversion and voltage regulation within the spacecraft electrical power system. It conditions electrical energy harvested from photovoltaic arrays, thermoelectric generators, RF harvesters, and radioisotope power sources through DC/DC converters and regulation circuits, ensuring compatibility with the spacecraft power bus and onboard loads. As the core component of PMAD, there have been many studies conducted around it to optimize its functionality. Cheng et al. [108] proposed a 4 kW hybrid-controlled buck–boost converter topology that achieves wide-range voltage conversion, smooth mode switching, and high conversion efficiency through component reuse and optimized control strategies. Zhang et al. [109] proposed a novel hybrid power-control strategy for a modular three-port converter (TPC)-based satellite power system. These studies improve power-conversion efficiency, enhancing power-flow flexibility, and facilitating the integration of multiple energy sources, thereby supporting the development of more autonomous and resilient spacecraft power systems.
2. Battery Management System (BMS): The Battery Management System supervises the operation of energy storage units by monitoring battery voltage, current, temperature, state of charge (SOC), and state of health (SOH). In addition, the BMS provides charge–discharge control, cell balancing, and protection functions to ensure safe and reliable battery operation throughout the mission lifetime. Jiménez et al. [110] presents a new BMS concept for spacecraft with enhanced performance, with not only monitoring and measurement functionalities, but also parameter calculation—such as estimations of the state of charge (SOC) and the state of health (SOH)—and cell balancing, in order to enlarge the lifespan of spacecraft batteries by providing active battery management. They also mentioned the shortage of research on spacecraft BMS. Therefore, more related research is expected.
3. Power Distribution Unit (PDU): PDU distributes regulated electrical power from the spacecraft electrical power system to onboard subsystems and payloads. It supports switched, unswitched, and pulsed power outputs, while providing command, telemetry, and fault-isolation functions to ensure reliable spacecraft operation [111].
4. Primary and Secondary Bus Converters: Bus converters are used to transfer power between different voltage buses within the spacecraft. Typically, a high-voltage primary bus collects energy from generation sources, while secondary buses provide regulated voltages required by specific loads. These converters improve power transmission efficiency and enable flexible system integration.
5. Telemetry and Protection Circuits: Telemetry and protection circuits continuously monitor critical electrical parameters such as voltage, current, power, and temperature. They provide real-time health information to the spacecraft control system and implement protection functions against overvoltage, overcurrent, short circuits, and other abnormal operating conditions.

The practical deployment of energy harvesting technologies depends not only on power generation capability but also on the characteristics of onboard energy storage and PMAD systems. In LEO missions, photovoltaic systems require substantial energy storage capacity to sustain operation during frequent eclipse periods, whereas GEO satellites experience reduced storage demands due to longer sunlight exposure. RF energy harvesting typically produces intermittent and low-power outputs, making efficient energy buffering and power conditioning essential regardless of orbital regime. TEGs provide supplementary power that depends on available temperature gradients and generally require storage units and PMAD circuits to stabilize output fluctuations. In contrast, RTGs deliver continuous power independent of solar illumination and are therefore particularly suitable for deep-space missions, although PMAD systems remain necessary for voltage regulation and power distribution. Consequently, the suitability of each energy harvesting technology is strongly influenced by the combined constraints of orbital environment, storage capacity, and power management architecture.

## 4. The Impact of Space Environment

**Figure 6:**
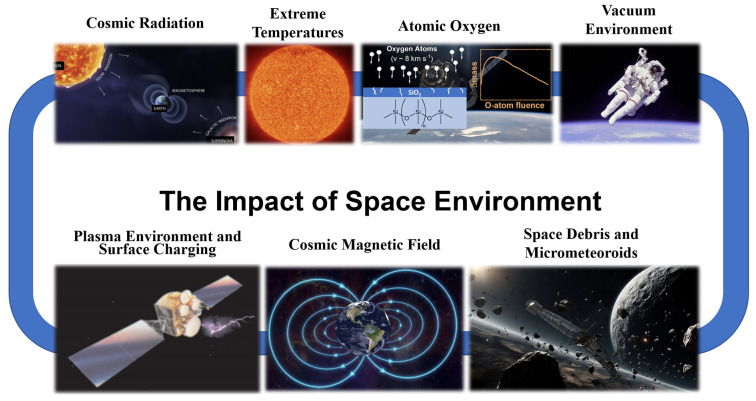
Effect of space environment on satellite.

The space environment is extremely harsh and there are multiple physical field coupling situations. Radiation, heat, vacuum, and plasma effects will affect the lifespan and performance of energy harvesting devices. Therefore, the design of space energy harvesting systems needs to consider environmental adaptability and long-term reliability characteristics. Table 4 shows the dominant environmental stress factors for different space energy harvesting technologies. Figure 6 shows the extreme environment of space.

### 4.1. Cosmic Radiation

Space radiation environment consists of solar energetic particles (SEPs) (10^6^–10^10^ eV/nucleon), galactic cosmic rays (GCRs) (10^8^–10^20^ eV/nucleon), energetic particles trapped in the South Atlantic anomaly region, and energetic electrons in radiation belts. Radiation effects on electronics can be classified into the following two classes: those caused by the total accumulated radiation dose over the life of a mission and those caused by single-event effects [112]. The materials of satellite solar panels need to have radiation resistance performance. High-energy particles include protons, electrons, and heavy ions [113]. In low Earth orbit, the radiation field is mainly SEPs, among which 90–95% are protons, 5–8% are helium nuclei, and 1% are heavier nuclei. In deep space, high-energy GCRs are the main part of the radiation field, and their composition is similar to that of solar energetic particles [114,115]. These high-energy particles can penetrate the satellite shell and directly damage internal components, such as displacement damage, internal charging, etc., making the satellite suffer irreversible damage. Radiation-induced degradation remains one of the primary lifetime-limiting factors for space photovoltaic systems. A study on GaAs solar cells operating in different Earth orbits revealed substantial variations in power degradation depending on the radiation environment. For a 15-year mission, proton-induced Pmax losses were estimated to be 51%, 31%, and 5% for MEO (10,000 km), MEO (20,000 km), and GEO, respectively, while electron-induced losses reached 36%, 40%, and 34%. These results indicate that electron irradiation can contribute comparably to, or even exceed, proton-induced degradation in certain orbital regimes. Consequently, both proton and electron radiation environments should be considered when evaluating long-term photovoltaic performance and shielding requirements [116].

### 4.2. Extreme Temperatures

Among the phenomena unfavorable to spacecraft, temperature-induced anomalies account for 11% [113]. During a solar eclipse, the temperature of the spacecraft’s surface fluctuates under direct sunlight, ranging from below −150 °C to above +150 °C. In low Earth orbit, the heat from outer space and the cold from the cold and dark areas will lead to obvious temperature changes. There are obvious thermal gradients in orbital spacecraft, which cause problems such as interface delamination, fatigue crack propagation, and mechanical failures. The temperature changes in geosynchronous orbit and deep space probes are relatively slow, and only the factors of the long-term operating environment need to be considered during design.

### 4.3. Atomic Oxygen

Atomic oxygen is formed in the low Earth orbital environment (LEO) by photo dissociation of diatomic oxygen by short wavelength (<243 nm) solar radiation which has sufficient energy to break the 5.12 eV O_2_ diatomic bond in an environment where the mean free path is sufficiently long (~108 m) that the probability of reassociation or the formation of ozone (O_3_) is small. Spacecraft impact the atomic oxygen resident in LEO with sufficient energy to break hydrocarbon polymer bonds, causing oxidation and thinning of the polymers due to loss of volatile oxidation products. Mitigation techniques, such as the development of materials with improved durability to atomic oxygen attack, as well as atomic oxygen protective coatings, have been employed with varying degrees of success to improve durability of polymers in the LEO environment. Atomic oxygen can also oxidize silicones and silicone contamination to produce non-volatile silica deposits. Such contaminants are present on most LEO missions and can be a threat to performance of optical surfaces Hooshangi et al. [117]. Study the damage of AO to aerospace satellite polymers, and AO also has an impact on spacecraft together with the vacuum environment. Harty and Gurnee [118,119] studied the impact of AO on spacecraft outgassing and found that AO exposure may change the outgassing parameters of some materials, but there are still differences in the response between materials, and the potential mechanism needs to be further studied.

### 4.4. Vacuum Environment

The atmospheric pressure in low Earth orbit is typically 10^−7^–10^−9^ Pa [120]; spacecraft will encounter situations such as gas escape, material evaporation, etc. Extremely low air pressure leads to a large amount of gas escape; when the internal pressure is extremely low (less than 10^−4^ Pa), internal gas escape may also be triggered. The released volatile components will redeposit on adjacent low-temperature surfaces, pollute the solar panels and reduce the power generation efficiency. Moreover, there is no oxide film protection in a vacuum, which increases the risk of cold welding of metal contacts and damages the reliability of the mechanical structure.

### 4.5. Plasma Environment and Surface Charging

Plasma environment is a common material state in the universe, which contains neutral particles and charged particles. When charged particles attach to the surface of a satellite, charge accumulation may occur. Geostationary orbit satellites often accumulate negative electricity at the kilovolt level (that is, surface charging and discharging phenomenon). This situation may lead to problems such as electrostatic discharge (ESD), electromagnetic pulse (EMP) interference, power loss or short circuit of solar panels, material degradation, and charge pollution [112]. According to the NASA manual, spacecraft charging remains a significant threat to satellite operation. Analysis of 11 years of DMSP observations revealed that satellites traversing auroral regions can experience surface potentials as low as −2000 V during intense electron precipitation events [121]. While low-Earth orbit spacecraft are mainly affected by auroral electron precipitation and plasma wake effects, geosynchronous spacecraft are exposed to severe surface charging caused by energetic magnetospheric electrons, with spacecraft potentials frequently exceeding −5 kV during geomagnetic storms and substorms [122]. This situation can be prevented by coating a conductive coating on the dielectric or by carrying out active plasma control.

### 4.6. Cosmic Magnetic Field

The height is reduced and the geomagnetic field strength is relatively strong. The near-Earth orbit is the main region affected by the geomagnetic force [123]. Space magnetic fields influence spacecraft operation through attitude perturbations, induced electromagnetic effects, and interactions with charged particles in the space plasma environment. Although magnetic fields generally do not directly degrade energy-harvesting devices, they can indirectly affect spacecraft power systems by modifying charged-particle transport, increasing surface charging risks, and contributing to radiation-induced degradation of electronic components and solar cells.

### 4.7. Space Debris and Micrometeoroids

Space debris poses a threat to satellites. It is divided into the following three categories: small (less than 1 cm), medium (1 to 10 cm), and large (more than 10 cm). Collisions of medium and large debris are relatively few, and they can be monitored and avoided; the impact of small debris is the most intensive. Hypervelocity collisions can produce plasma clouds, which may damage the electronic equipment of satellites. Usovik [124] evaluated methods to prevent space debris from damaging satellites and provided guidance for satellite design.

Space energy harvesting systems are rarely subjected to a single environmental stressor; instead, their long-term performance is governed by the synergistic interaction of radiation, thermal cycling, atomic oxygen erosion, and spacecraft charging. For example, photovoltaic arrays operating in low Earth orbit (LEO) may experience more than 5000 thermal cycles per year due to repeated transitions between sunlight and eclipse, while simultaneously being exposed to atomic oxygen fluences exceeding 10^21^ atoms cm^−2^ over mission lifetimes. Radiation-induced defects generated by high-energy electrons and protons can reduce carrier lifetimes and cell efficiency, whereas thermal cycling promotes crack initiation and interconnect fatigue. These degradation mechanisms often interact, as radiation-induced material embrittlement accelerates thermally driven crack propagation. GaAs solar cells may suffer power losses of approximately 10–30% over typical 10–15-year missions, with even higher degradation possible in medium-Earth orbits due to enhanced particle radiation exposure. Similar synergistic effects are observed in thermoelectric systems, where repeated thermal cycling increases interfacial thermal resistance and mechanical fatigue, thereby reducing power output despite stable thermoelectric material properties. Consequently, the operational lifetime of spacecraft energy systems is frequently determined not by a single environmental factor but by the cumulative and coupled effects of multiple stressors acting simultaneously.

## 5. Applications of Different Orbital Satellites

The altitude of low Earth orbit (LEO) is 160–2000 km, the altitude of geosynchronous orbit (GEO) is 35,786 km, the orbit of deep space probes exceeds 1.5 × 10^6^ kilometers, and different orbits have different requirements for spacecraft.

### 5.1. Low Earth Orbit Satellites

The density of low Earth orbit (LEO, roughly 160 to 2000 km) satellites is relatively high. Spacecraft operating in LEO may range from CubeSats with power demands of only a few watts to large communication or Earth-observation satellites requiring several kilowatts of continuous power. Low Earth orbit satellites have a relatively short orbital period; the energy system needs to adapt to the changes in the solar activity cycle. The Earth’s day–night cycle is 90 min, so the solar panels need to have good cyclic stability and resistance to thermal shock.

In the orbital environment, solar energy belongs to the main energy source. Multijunction solar cells based on silicon (Si) and group III/V (III/V) are widely used. Low Earth orbit (LEO) is in a high atomic oxygen (AO) and plasma environment, and the surface of solar cell and packaging materials need to have the performance of resisting atomic oxygen erosion and charge cycling. Low Earth orbit satellites (LEO satel) need high specific power (W·kg^−1^), and lightweight design and deployable array structure have become key technical points. Therefore, developing CIGS or perovskite solar cells to replace multi-junction cells is a very promising choice.

In recent years, the trend of small satellite and constellation deployment has been more rapid, and multi-source energy coordination (such as auxiliary heat power recovery system) has gradually become a hot research matter. The collection of waste heat from solar panels is an excellent auxiliary energy source for satellites. New research is being conducted around this topic.

### 5.2. Geostationary Orbit Satellite

Geosynchronous orbit satellites (about 35,786 km) are used for communication broadcasting, meteorological monitoring, and data relaying. Low Earth orbit satellites are different. Geosynchronous orbit satellites are not affected by atomic oxygen and can operate for a long time under high-energy electromagnetic radiation. The radiation effect is relatively critical for the life of the energy system.

GEO satellites are often surrounded by solar radiation, and some solar eclipse situations occur at the equinox. The solar system needs long-term radiation stabilization and power degradation control. Also, III/V multi-junction solar cells have the characteristics of high efficiency and radiation resistance and have become the mainstream situation.

GEO satellites usually operate with high power (several kilowatts or higher) and have more strict requirements for power management and thermal control systems. This also means that thermoelectric generators can use satellite waste heat to generate electricity. With the growth of communication demand, components with high power density and conversion efficiency have become key in the development of geostationary orbit energy systems.

### 5.3. Deep Space Probe

Deep space missions (such as to the Moon, Mars, etc.) are far from Earth’s orbit. The energy there is limited by the attenuation of solar radiation. As the distance from the Sun increases, the irradiance per unit area decreases with the square of the distance, which makes the output of traditional photovoltaic systems decrease significantly.

In the near-solar system region, such as inside the orbit of Mars, efficient solar panels are feasible, but large-scale deployment is needed to make up for the insufficient radiation exposure. In the far-solar system region, such as Jupiter and beyond, radioisotope thermoelectric generators (RTGs) are the main energy sources. This device converts the heat generated by radioactive decay into electricity, providing long-term stable power.

Table 5 presents the recommended energy harvesting strategies for different orbital environments. Solar photovoltaic systems remain the dominant power source in both LEO and GEO missions due to their high maturity and proven flight heritage. In contrast, the relevance of RTGs increases significantly for deep-space exploration missions because solar irradiance decreases rapidly with distance from the Sun. Thermoelectric generators (TEGs) are mainly considered supplementary energy sources across all orbital regimes, while RF energy harvesting is currently limited to low-power auxiliary applications due to its relatively low-power density and low technology readiness level.

Table 6 compares the key performance metrics and technology readiness of major space energy harvesting technologies. Solar photovoltaic systems exhibit the highest combination of power output, specific power, technological maturity, and flight heritage, making them the primary power source for most spacecraft. RTGs provide exceptional long-term reliability and radiation tolerance and are therefore indispensable for deep-space missions. In contrast, TEGs and RF energy harvesting systems generally generate only milliwatt-level power and are better suited as auxiliary power sources for low-power electronics, sensors, and health-monitoring systems. Although these emerging technologies offer unique advantages, further in-orbit validation is required before large-scale deployment in future spacecraft energy systems.

## 6. Challenges and Outlook

### 6.1. Challenges

Based on the previous discussion in the article, we can conclude that there are still the following challenges in current satellite energy harvesting technology:

(1) Reliability: Emerging technologies such as perovskite and flexible photovoltaic devices often exhibit promising performance in laboratory environments but still face challenges related to radiation-induced degradation, thermal cycling, atomic oxygen erosion, and packaging reliability under long-duration space exposure. Furthermore, high-performance space-grade materials remain costly and difficult to manufacture at large scale.
(2) Economics: Beyond technical performance, practical deployment is strongly influenced by manufacturing scalability, launch mass constraints, system complexity, and mission cost. Technologies with excellent laboratory performance may still face significant barriers to adoption if their fabrication processes, qualification requirements, or operational costs are incompatible with spacecraft mission economics.
(3) On-orbit verification: The serious lack of on-orbit flight test data for emerging technologies; the lack of unified test standards and evaluation systems for space energy harvesting devices; the high cost and long cycle of on-orbit verification.

### 6.2. Outlook

Aiming at the above challenges, the future development directions of space satellite energy harvesting technology are proposed:

(1) Future space energy systems may employ self-healing photovoltaic materials capable of mitigating radiation-induced defects and microcrack propagation, thereby extending operational lifetime in harsh space environments. In addition, multifunctional structural-energy materials that simultaneously provide load-bearing, power-generation, and energy-storage functions could significantly reduce spacecraft mass and improve system-level energy efficiency.
(2) Development of new materials: In micro and deep space missions, specific power (W·kg^−1^) is a key point, so it is necessary to develop ultra-thin III/V group, flexible perovskite stacks. At the same time, the development of metamaterials, new thermoelectric materials, and dynamic radioisotope power systems is also crucial.
(3) Future spacecraft may adopt highly integrated energy modules that combine energy harvesting, energy storage, power management, and health-monitoring functions within a single package. Such architectures can reduce system complexity, improve reliability, optimize power utilization, and support autonomous operation of next-generation satellite constellations and deep-space missions.

## 7. Summary

This review comprehensively summarizes the current status, performance characteristics, environmental adaptability, and mission applicability of major space energy harvesting technologies, including photovoltaic power generation, RF energy harvesting, thermoelectric generators (TEGs), and radioisotope thermoelectric generators (RTGs). The analysis demonstrates that different technologies occupy distinct roles within spacecraft power architectures and should be evaluated according to mission requirements, orbital environments, power demand, and technology maturity.

Among the reviewed technologies, photovoltaic power generation remains the dominant energy source for most contemporary spacecraft. Silicon solar cells continue to be widely adopted in cost-sensitive LEO missions, while III–V multi-junction photovoltaic devices represent the most mature and reliable solution for high-performance Earth-orbiting and deep-space missions due to their high conversion efficiency, radiation tolerance, and extensive flight heritage. RTGs remain the only fully proven long-duration power source for missions operating in regions where solar irradiance is insufficient to sustain conventional photovoltaic systems.

Thermoelectric energy harvesting offers opportunities for waste-heat recovery and auxiliary power generation, although its practical contribution is often constrained by the limited temperature gradients available on spacecraft. RF energy harvesting provides an alternative approach for powering distributed ultra-low-power sensors and monitoring nodes; however, the harvested power typically remains several orders of magnitude lower than the power requirements of most spacecraft systems. Consequently, RF harvesting is currently better regarded as a supplementary technology rather than a primary spacecraft power source.

The review further highlights substantial differences in technology readiness among the various approaches. Conventional photovoltaic systems and RTGs have accumulated extensive flight experience and have reached high levels of technological maturity, whereas flexible photovoltaics, perovskite solar cells, advanced thermoelectric systems, and RF energy harvesting technologies remain at earlier stages of development and require further validation under long-term space environmental conditions. Key challenges for future deployment include radiation resistance, packaging reliability, thermal-mechanical durability, manufacturability, and large-scale space qualification.

## Figures and Tables

**Table 2:** Comparison of electromagnetic RF power generation.

References	Year	Service Frequency	RF-DC Efficiency	Output Voltage	Characteristic
[80]	2023	5.8 GHz	75.2% (90 μW/cm^2^)	9.1 V (10 elements)	A smooth regular array extension antenna composed of 5 × 2 microstrip antennas is designed to enhance the receiving power level at different angles, improve the intensity of low-receiving-power density, and achieve high voltage and high current.
[67]	2024	11.5–12.5 GHz	20% (−10 dBm)	1.88 V	Maintains robustness to variations in received power and incident angle (from −65° to 65°), while supporting broadband operation across the 11.5 GHz to 12.5 GHz frequency range.
[69]	2025	2–18 GHz	27% (−15 dBm)	>3.7 V	At low input power (below −10 dBm), the output DC voltage across the 2–18 GHz frequency range consistently exceeds 3.7 V. The average power conversion efficiency is 30% at low input power (below −15 dBm). The power conversion efficiency of the input power is less than −5 dB in all working frequency ranges, exceeding 45%. The power conversion efficiency exceeds 40% and the power density reaches 305 μW/cm^2^ within the 0–360° angle range.
[68]	2025	3.6–6.45 GHz	59.82% (9 dBm)	1.9 V	Coverage in the 3.6–6.45 GHz frequency band.
[71]	2025	0.9 GHz, 1.8 GHz	68.94% (−5 dBm)	0.606 V	A novel single-pole antenna with a defective ground structure (DGS) has been developed.
[81]	2026	2.45 GHz	71% (5 dBm)	1.572 V	The system implements four different radiation beam modes: three multi-beam modes and one high-gain single-beam mode to achieve wide-angle coverage.

**Table 4:** Dominant environmental stress factors for different space energy harvesting technologies.

Technology	Major Environmental Stressors	Primary Degradation Mechanisms
Photovoltaic Cells	Radiation, thermal cycling, atomic oxygen	Semiconductor damage, encapsulation degradation, interconnect failure
RF Energy Harvesters	Thermal cycling, charging, radiation	Rectifier degradation, impedance mismatch, ESD damage
Thermoelectric Generators	Thermal cycling, mechanical stress, radiation	Interface degradation, solder fatigue, increased thermal resistance
Flexible Harvesters	Atomic oxygen, UV radiation, thermal cycling	Polymer erosion, delamination, cracking

**Table 5:** Energy collection schemes for different orbits.

Orbit	Dominant Energy Source	Auxiliary Options	Major Environmental Stressors
LEO	Solar PV (TRL 9)	RF harvesting (TRL 2–4), TEG (TRL 3–5)	Atomic oxygen, thermal cycling, radiation, surface charging
GEO	Solar PV (TRL 9)	RF harvesting (TRL 2–4), TEG (TRL 3–5)	Electron radiation, deep dielectric charging, thermal cycling
Lunar Missions (~1 AU)	Solar PV (TRL 9)	TEG (TRL 3–5)	Lunar dust, thermal extremes, radiation
Mars Missions (~1.5 AU)	Solar PV (TRL 9), RTG (TRL 9)	TEG (TRL 3–5)	Dust storms, radiation, low temperatures
Inner Solar System Deep Space (≤5 AU)	Solar PV (TRL 9), RTG (TRL 9)	TEG (TRL 3–5)	Solar particle events, cosmic radiation, thermal extremes
Outer Solar System (>5 AU)	RTG (TRL 9)	Advanced nuclear systems	Cosmic rays, extreme cold, micrometeoroids

**Table 6:** Comparison of key performance metrics and technology readiness of space energy harvesting technologies.

Technology	Typical Power Output	Specific Power (W/kg)	Radiation Tolerance	Lifetime	TRL	Flight Heritage
Solar PV	10 W–100 kW	50–300	Moderate–High	10–15+ years	9	Extensive
TEG	1 mW–500 mW	0.1–10	High	10–20+ years	3–5	Limited
RF Energy Harvesting	1 μW–10 mW	<1–10	High	Not well-established	2–4	Very limited
RTG	100 W–kW	2–10	Very High	10–30+ years	9	Extensive

## Data Availability

This study did not generate any new datasets. All data analyzed are from publicly available sources, as cited in the manuscript.
